# First song descriptions of some Anatolian species of Tettigoniidae Krauss, 1902 (Orthoptera, Ensifera)

**DOI:** 10.3897/zookeys.369.5864

**Published:** 2014-01-13

**Authors:** Deniz Şirin, Mehmet Sait Taylan, Abbas Mol

**Affiliations:** 1 Department of Biology, Faculty of Art and Science, University of Namık Kemal, Tekirdağ, Turkey; 2 The Society of Anatolian Speleology Group (ASPEG), Serpil Sk., Yıldız Apt. 14/A, Kavacık, Beykoz, İstanbul, Turkey; 3 Guzelyurt Vocational School, Aksaray University, Aksaray, Turkey

**Keywords:** Acoustic analysis, Tettigoniinae, Bradyporinae, Saginae, Anatolia

## Abstract

Fourteen endemic and two sub-endemic species belonging to three subfamilies of Tettigoniidae (Tettigoniinae, Bradyporinae and Saginae) were sampled during field trips throughout the different ranges of Anatolia between the years of 2004 and 2013. Acoustic parameters of these 16 species affiliated to 8 genera (*Anterastes*, *Apholidoptera*, *Gampsocleis*, *Parapholidoptera*, *Pezodrymadusa*, *Psorodonotus*, *Bradyporus* and *Saga*) have been described for the first time in this study. Acoustical analysis showed that song characters are species-specific in the genera *Saga* and *Psorodonotus*. On the other hand, we could not find big differences among species of the genus *Pezodrymadusa* and *Parapholidoptera castaneoviridis* species-group.

## Introduction

Orthoptera is one of the most well-known acoustically active insect orders (Heller 2006). The taxa of Tettigoniidae produce specific songs, which allow the recognition, location and selection of conspecific mating partners (e.g., Walker 1964, Heller 1988, Ewing 1989, Heller 1990, Robinson and Hall 2002, Heller 2006). Almost all species have a specific song structure, hence the useful and functional taxonomic character (Heller 2006) which allows the discrimination of morphologically similar species (Ingrisch 1991, Ragge and Reynolds 1998, Heller et al. 2004, Kolics et al. 2012). On the other hand, some genera of *tettigoniids*, such as *Parapholidoptera* (Heller 1988) and *Eupholidoptera* (Çıplak et al. 2009) exhibit, characteristic songs and uniform intrageneric song patterns.

The researchers of the last century were able to document many of the singing Orthoptera that are distributed in certain areas, such as North America (Walker and Moore 2004), Eastern United States (Alexander 1956) and Europe (Heller 1988, Ragge and Reynolds 1998). The history of studies that include song analysis in Anatolian Orthoptera began with Stumpner and Helversen (1992) for Caelifera and Heller (1988) for Ensifera. Up to now, as many as 55 songs of endemic *tettigoniids* from Anatolia have been already described and these studies can be divided into three main categories: (i) single species song description (Çıplak and Heller 2001, Çıplak et al. 2002, Çıplak and Heller 2005, Çıplak et al. 2006, Sevgili et al. 2012a); (ii) song descriptions of species-group and/or groups in a genus (Heller 2004, Sevgili 2004, Heller and Sevgili 2005, Heller et al. 2006, Sevgili et al. 2006, Çıplak et al. 2009, Heller et al. 2008, Heller and Korsunovskaya 2009, Sevgili et al. 2010, Kaya et al. 2011, Kaya and Çıplak 2011, Kaya et al. 2012, Chobanov et al. 2013); and (iii) songs of orthopteran species in a certain area (Sevgili et al. 2011). Moreover, a huge part of Anatolian *tettigoniids* has not been studied with regards to the song characteristics until now.

The family Tettigoniidae Krauss (1902) is the largest family of the Orthoptera and it displays species richness in Anatolia of about 360 taxa (Karabağ 1958, Çıplak et al. 2002, Ünal 2013a). More than 60% of Tettigoniidae taxa (e.g. Karabağ 1958, Ünal 2002) recorded from Turkey are endemic to Anatolia (Çıplak et al. 1993, Çıplak and Demirsoy 1995, Çıplak et al. 2002). A possible explanation for the richness of the Tettigoniidae species and its high endemism rate in Anatolia is that this region is one of the most important refugium in Palearctic (Hewitt 1996, Çıplak 2003, Şirin et al. 2010). However, the studies on the lineages represented in this peninsula are still far from explaining this phenomenon.

In the present study, we aim (i) to obtain the first ever records of song characteristics of 14 endemic and 2 sub-endemic species belonging to 8 genera (*Anterastes*, *Apholidoptera*, *Gampsocleis*, *Parapholidoptera*, *Pezodrymadusa*, *Psorodonotus*, *Bradyporus* and *Saga*) from different parts of Anatolia and (ii) to understand the relation between the distribution and song diversity of the species under discussion.

## Methods

### Specimens collecting

In the present study, 16 species of 8 genera belonging to three different subfamilies of Tettigoniidae (Tettigoniinae, Bradyporinae and Saginae) were sampled during field trips throughout the different ranges of Anatolia between 2004 and 2013. Male calling songs were recorded in the field or in laboratory from live animals. Then, the recorded specimens were collected, labelled and deposited in 96% ethyl alcohol. Specimens examined in this study are deposited Aksaray University Central Research Laboratory, Entomological Museum, ASUBTAM (Aksaray/Turkey), Namık Kemal University, Department of Biology, Entomological Museum NKUEM (Tekirdağ, Turkey), and the personal collection of M.S. Taylan.

### Song recording and analysis

Song recordings of collected animals were made in the field and laboratory. All song records were carried out by TASCAM DR-100 recorder using Philips-SBC ME 570 condenser microphone (frequency response flat up to 18 kHz) and SONY RECORDER with a shotgun microphone (the upper frequency limit was 15 kHz). The microphone was kept about 5–15 cm away from the calling male. The male songs were analyzed with custom-designed software (W. Schulze) developed in LabVIEW 7 (National Instruments, Austin, TX, USA) and Turbolab 4.0 (Stemmer AG). The traditional Ensifera song terminology (Heller 1988, Ragge and Reynolds 1998, Heller et al. 2006) is slightly modified to describe the songs of *tettigoniids* more accurately.

**Figure 1.**
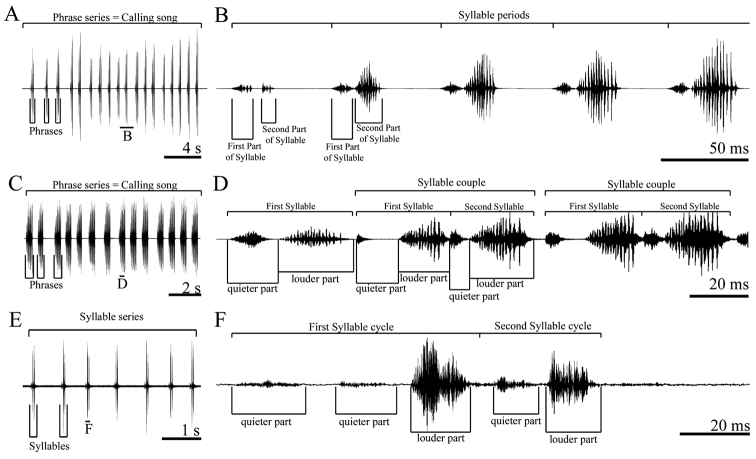
Terminology for three complex song types in studied *tettigoniids*. *Parapholidoptera bolkarensis* – total song (**A**) and one phrase in detail (**B**) *Pezodrymadusa kurmana* – total song (**C**) and several syllable couples in detail (**D**) *Psorodonotus davisi* – syllable series (**E**) and syllable cycles in detail (**F**).

The following terms were used: *Calling song*, song produced by an isolated male; *phrase*, a first-order assemblage of syllables; *syllable*, the song produced by one opening-closing movement cycle of the tegmina; *syllable interval*, time from end of last impulse to beginning of first impulse of the next syllable; *impulse*, a simple undivided transient train of sound waves; *pulse*, a long train of sound waves, resulting from the fusion of several impulses (Figure 1). In song descriptions (minimum value-maximum value (mean value ± standart deviation)), seconds (s) or milliseconds (ms) were used for duration/intervals.

## Results

### Tettigoniinae Krauss, 1902

#### Anterastes tolunayi

Karabağ, 1951

http://species-id.net/wiki/Anterastes_tolunayi

##### Distribution.

**Figure 2.**
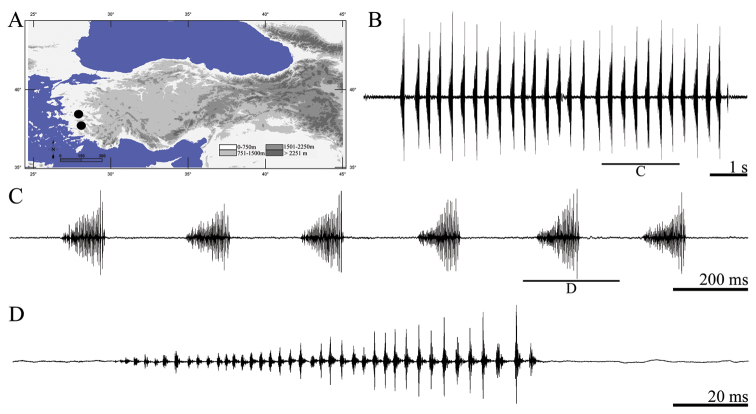
Distribution map (**A**) and male calling song of *Anterastes tolunayi* (**B** one complete phrase **C** a group of syllables and **D** one complete syllable).

*Anterastes tolunayi* has been recorded from Aydın and İzmir provinces of Turkey (Figure 2a) (Karabağ 1951, 1958, Çıplak 2004).

##### Song recording.

Males collected from İzmir, Bozdağ, Günalan yaylası-millik mevkii, 38°21.110'N, 28°06.245'E, 1545 m, 15.VI.2010 (by D. Şirin) and calling song recorded from two males at 26 °C in laboratory (by D. Şirin).

##### Description of song.

Eight records from two males were analyzed. The calling song consists of sequences of polysyllabic phrases of different duration (Figure 2b, 2c), each of the phrases repeated regularly and lasting 94–150 ms (124 ± 0.03). Amplitudes of syllables getting louder from the first syllable to last one (Figure 2d). Therefore general song type of the phrase is typical crescendo. The number of syllables within 100 ms is 29–40 (34.62 ± 2.21) (Figure 2d). Syllable duration varies between 2 and 5 ms (3.67 ± 0.34) with an interval of 0–1 ms (0.56 ± 0.06).

#### Apholidoptera pietschmanni

(Ebner, 1912)

http://species-id.net/wiki/Apholidoptera_pietschmanni

##### Distribution.

**Figure 3.**
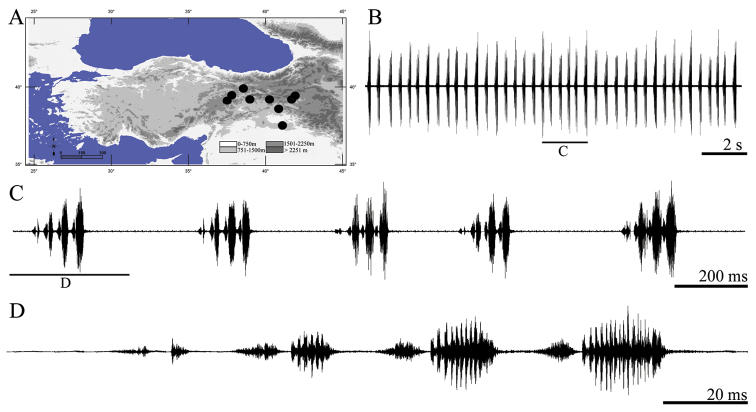
Distribution map (**A**) and male calling song of *Apholidoptera pietschmanni* (**B** sequences of phrases **C** a group of phrases and **D** one complete phrase).

Turkey and Iraq (Figure 3a) (Karabağ 1958, Sevgili and Çıplak 2000, Ünal 2006).

##### Song recording.

Male specimens collected from Turkey, Erzincan, Kemaliye, Ocak köyü, 39°08.732'N, 38°35'.296'E, 1485 m, 3.VII.2012 (by D. Şirin & A. Mol) and calling song recorded from one male at 32 °C in laboratory (by D. Şirin).

##### Description of song.

Eight records from one male were evaluated. The calling song consists of a series of regular phrases (Figure 3b) each of which lasting 138–168 ms (152 ± 0.01) and consisting of 3–5 (4.01 ± 0.37) syllables. Syllables generally consist two uneven parts (Figure 3c, 3d). The number of syllables in 100 ms is 2.5–3 (2.62 ± 0.02). The first syllable at the beginning of the phrase is quieter (lower amplitude) than other syllables (Figure 3c, 3d). Syllable duration varies between 17 and 36 ms (29.25 ± 4.15) with an interval of 0–13 ms (5.22 ± 1.78).

#### Gampsocleis recticauda

Werner, 1901

http://species-id.net/wiki/Gampsocleis_recticauda

##### Distribution.

**Figure 4.**
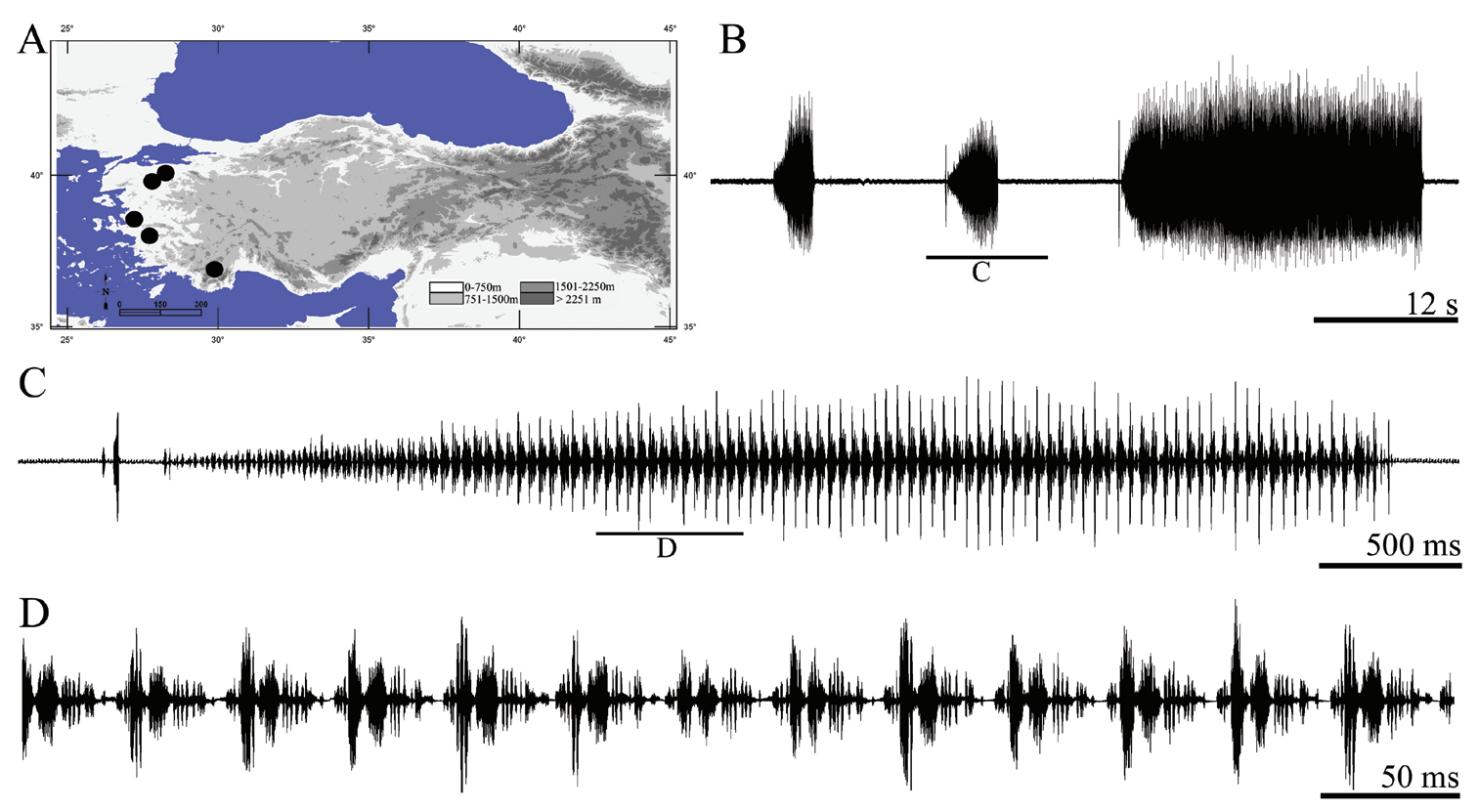
Distribution map (**A**) and male calling song of *Gampsocleis recticauda* (**B** a complete song **C** a complete phrase and **D** a group of syllables).

Endemic for Turkey – Western Anatolia (Figure 4a) (Karabağ 1958, Karabağ et al. 1971).

##### Song recording.

Male specimens collected from Turkey, Antalya, Elmalı, Bozöyük köyü-Uzunkarış Tepe arası, 36°43.509'N, 30°09.298'E, 1768 m, 9.VII.2008 (by D. Şirin & U. Şirin) and calling song recorded from two males at 33 °C in the field (by D. Şirin).

##### Description of song.

Total of the six records from two males was analyzed. The calling song consists of several phrases in different duration (Figure 4b). The phrases begin with thick pulse and continue with low intensity in the first part of the phrase. The following part of phrases consists of song elements with higher intensity (Figure 4c). Phrases duration varies between 3.53–25.95 s (11.32 ± 5.53). Syllable duration varies between 33 and 40 ms (36.72 ± 1.28) with an interval of 0–3 ms (1.52 ± 0.09). Oscillographic analyses showed that each syllable contains different number of parts which are divided by the very short interval (lower than 2 ms). First and last part of a syllable generally consist of 2–4 shorter elements (each of 1 ms), while middle part consists of two longer elements (each of 6–8 ms).

#### Parapholidoptera bolkarensis

Çıplak, 2000

http://species-id.net/wiki/Parapholidoptera_bolkarensis

##### Distribution.

**Figure 5.**
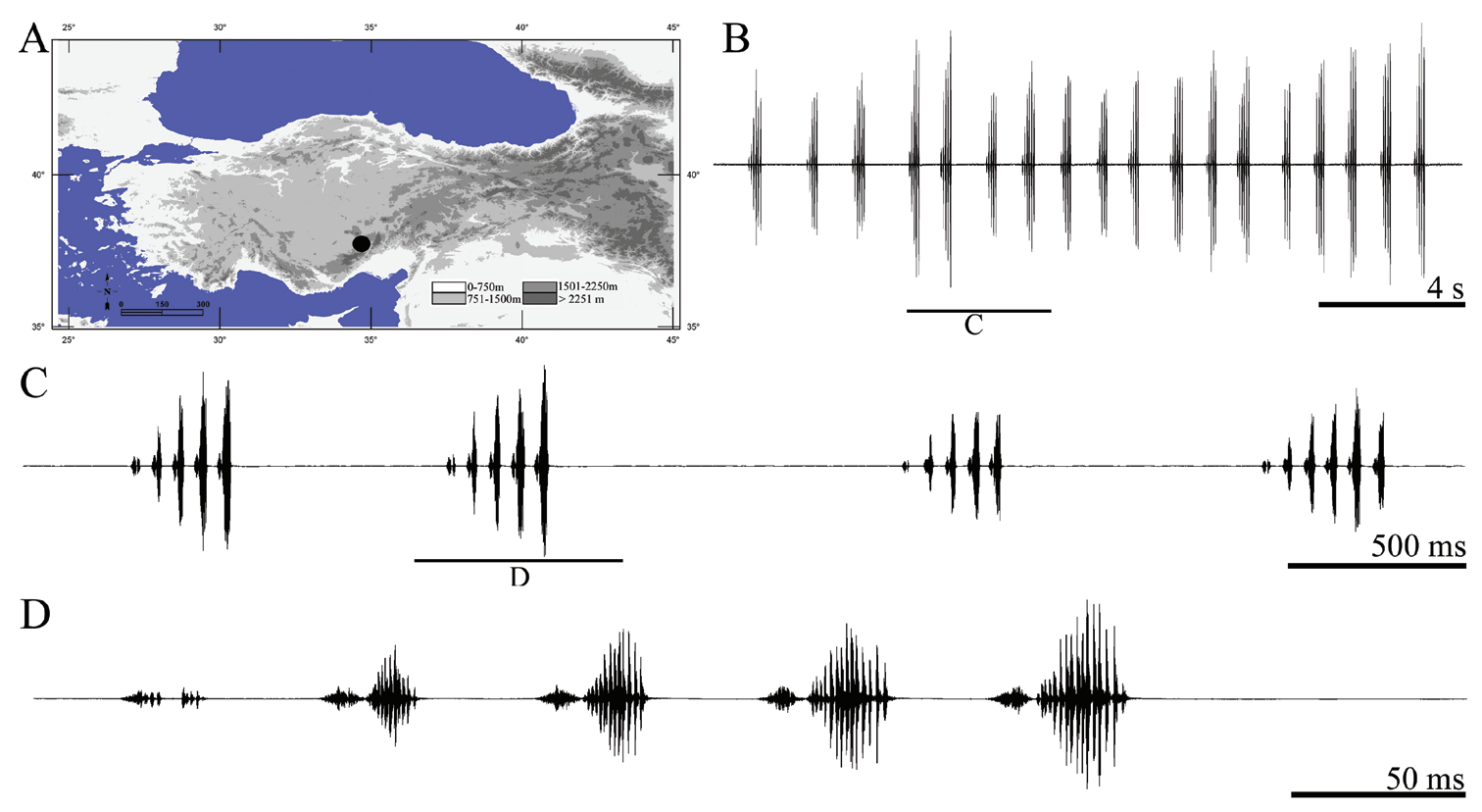
Distribution map (**A**) and male calling song of *Parapholidoptera bolkarensis* (**B** sequences of phrases **C** a group of phrases and **D** one complete phrase).

Endemic for Turkey, Bolkar Mountains (Figure 5a) (Çıplak 2000).

##### Song recording.

Male specimens collected from Turkey, Niğde, Ulukışla, Karagöl Yolu, Bolkar Dağları, 2285 m (type locality), 12.VIII.2011 (by M. S. Taylan, A. Aydın) and calling song recorded from two males at 25 °C in the field (by M. S. Taylan).

##### Description of song.

Total of the six records from two males was examined. The calling song consists of a series of regular phrases (Figure 5b) with an interval of 509–1259 ms (0.76 ± 0.10). Phrase durations vary between 219–346 ms (294 ± 0.04) and phrases consist of 4–6 (5.21 ± 0.54) syllables. The first and second syllables at the beginning of the phrase are quieter and shorter (having low amplitudes) than the following ones (Figure 5c). Syllable duration varies between 17 and 41 ms (30.74 ± 4.08) with an interval of 21–47 ms (28.52 ± 3.13). Oscillographic analyses showed that each syllable contains two parts. First part of syllables relatively short and consist of comprised song elements (Figure 5d). First parts generally last 8–13 ms (11.71 ± 1.16) and are followed by second part after an interval of 0–5 ms (1.22 ± 0.48). The second syllable part includes several high amplitudes elements (Figure 5d). These elements number is always 3–4 in first syllable and following respectively 9–10, 12–13, 13–15, 16–18 and 16–18 in last syllable. The second syllable part is much louder (except of the first syllable) and longer than the first part and duration varies between 9 and 27 ms (16.71 ± 3.76).

#### Parapholidoptera intermixta

Karabağ, 1961

http://species-id.net/wiki/Parapholidoptera_intermixta

##### Distribution.

**Figure 6.**
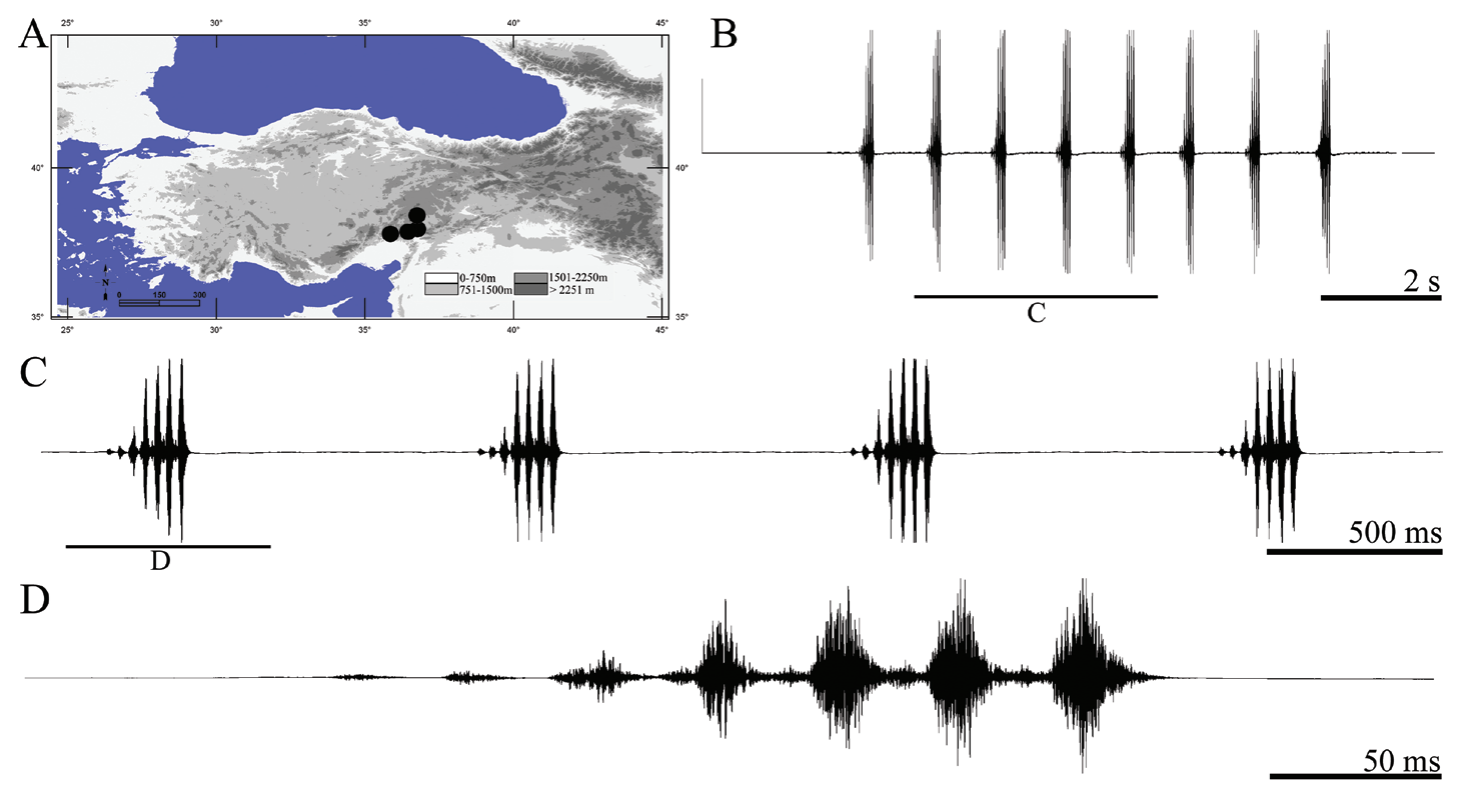
Distribution map (**A**) and male calling song of *Parapholidoptera intermixta* (**B** sequences of phrases **C** a group of phrases and **D** one complete phrase).

Endemic for Turkey, Binboğa-Mountains (Figure 6a) (Karabağ 1961, Çıplak 2000).

##### Song recording.

Male specimens collected from Turkey, Adana, Saimbeyli, Obruk Saksağan boğazı, 1410 m, 03.VII.2010 (by D. Şirin) and calling song recorded from five males at 28 °C in the field (by D. Şirin).

##### Description of song.

Ten records from five males were examined. The calling song consists of a series of regular phrases (Figure 6b) with an interval of 681–895 ms (810 ± 0.07). Phrase durations vary between 239–254 ms (246 ± 0.05) and phrases consist of 6–7 (6.12 ± 0.35) syllables. Syllables consisting of denser and hardly distinguishable impulses (Figure 6d). The first and second syllables at the beginning of the phrase are quieter and shorter (having low amplitudes) than the following syllables (Figure 6c, d). Syllable duration varies between 29 and 60 ms (38.59 ± 5.78) without any silent interval [except between the first and second syllables (6–12 ms)].

#### Parapholidoptera salmani

Çıplak, 2000

http://species-id.net/wiki/Parapholidoptera_salmani

##### Distribution.

**Figure 7.**
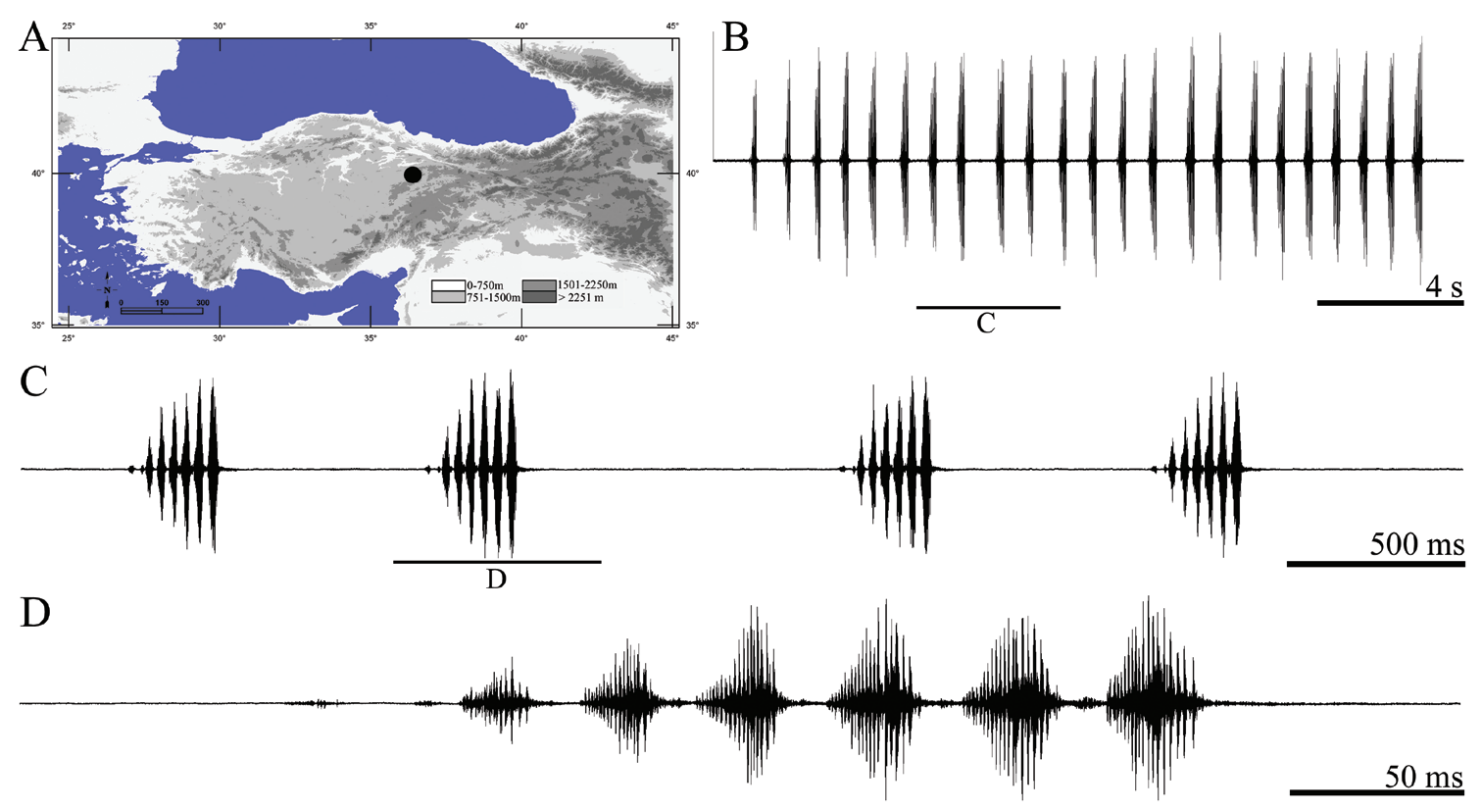
Distribution map (**A**) and male calling song of *Parapholidoptera salmani* (**B** sequences of phrases **C** a group of phrases and **D** one complete phrase).

Endemic for Central Anatolia and the Black Sea area of Turkey (Figure 7a) (Çıplak 2000, Ünal 2006).

##### Song recording.

Male specimens collected from Turkey, Tokat, Çamlıbel Geçidi, 1960 m., 02.VIII.2011 (by M.S. Taylan) and calling song recorded from three males at 30 °C in the field which is type locality of species (by M.S. Taylan).

##### Description of song.

Six records from three males were examined. The calling song consists of a series of regular phrases (Figure 7b) with an interval of 522–845 ms (612 ± 0.09). Phrase durations vary between 220–304 ms (265 ± 0.02) and phrases consist of 5–8 (6.35 ± 0.63) syllables. Syllables consist of several high amplitudes elements (Figure 5d). These elements number is always uncountable in first syllable, 9–13 in second syllable and 17–24 (generally 20–22) in following syllables. The phrase begins with 2–3 low amplitude syllables and the maximum intensity is usually reached between 3/8–3/5 of the phrase (Figure 7c, 7d). Syllable duration varies between 23 and 43 ms (35.01 ± 5.78) with an interval of 0–3 ms (2.21 ± 0.17).

#### Pezodrymadusa konowi

(Bolivar, 1899)

http://species-id.net/wiki/Pezodrymadusa_konowi

##### Distribution.

**Figure 8.**
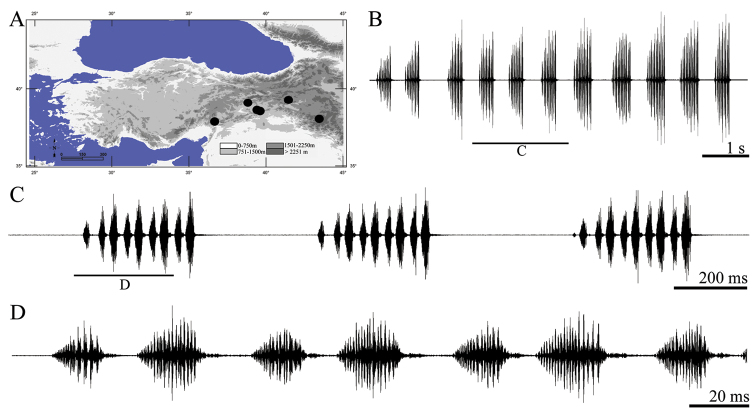
Distribution map (**A**) and male calling song of *Pezodrymadusa konowi* (**B** sequences of phrases groups **C** two complete phrase and **D** a group of syllable couples).

Endemic for Turkey – East Anatolia (Figure 8a) (Karabağ 1961, Sevgili et al. 2012b).

##### Song recording.

Male specimens collected from Turkey, Elazığ, Keban çıkışı 15 km, 38°42.790'N, 38°57.428'E, 1376 m, 03.VII.2012 (by D. Şirin & A. Mol), and calling song recorded from two males at 32 °C in laboratory (by D. Şirin).

##### Description of song.

Five records from two males were evaluated. The calling song consists of a series of irregular number of phrases (Figure 8b) with an interval of 297–615 ms (376 ± 0.09). Phrases are consisting of 9–11 (9.84 ± 0.99) syllables. The phrase begins with a quiet syllable (Figure 8c). Oscillographic analyses showed that each phrase involves two syllable couples (Figure 8d). Syllable couple duration varies between 53–67 ms (54.10 ± 1.97) with an interval of 5–19 ms (8.37 ± 2.17). First syllable in these couples lasts 19–25 ms (22.33 ± 1.67) and contains a louder beginning part [17–23 ms (21.12 ± 2.27)] and a quieter part [1–4 ms (1.97 ±0.78)]. First syllable in these couples is followed by a second syllable (except the first syllable in a phrase) after an interval of 6–12 ms (8.22 ± 1.63). Duration of the second syllable in these couples varies between 26–34 ms (28.57 ± 2.15).

#### Pezodrymadusa kurmana

(Ramme, 1939)

http://species-id.net/wiki/Pezodrymadusa_kurmana

##### Distribution.

**Figure 9.**
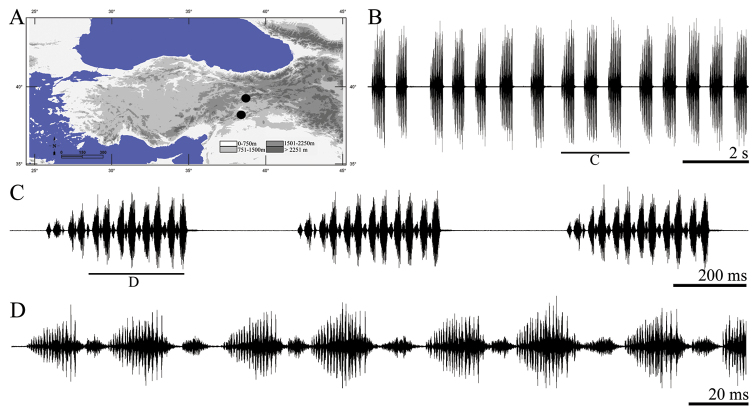
Distribution map (**A**) and male calling song of *Pezodrymadusa kurmana* (**B** sequences of phrases groups **C** three complete phrase and **D** a group of syllable couples).

Endemic for Turkey – East Anatolia (Figure 9a) (Karabağ 1961, Sevgili et al. 2012b).

##### Song recording.

Male specimens collected from Turkey, Malatya, Yeşilyurt, Gündüzbey–Adıyaman yolu, Bürücek Yaylası, 38°11.425'N, 38°19.102'E, 1862 m, 02.VI.2010 (by D. Şirin), Doğanşehir, Çığlık, Gürobası köyü üstleri, 38°05.138'N, 37°58.576'E, 1791 m, 04.VII.2012 (by D. Şirin & A. Mol) and calling song recorded from three males at 32 °C in laboratory (by D. Şirin).

##### Description of song.

Eight records from three males were evaluated. The calling song consists of a series of phrases (Figure 9b) with an interval 256–693 ms (392 ± 0.13). Phrases are consisting of 9–13 (10.40 ± 0.98) syllables. The phrase begins with a quiet syllable (Figure 9c). Oscillographic analyses showed that each phrase involves a few couples of syllables (Figure 9d). Syllable couple duration varies between 50–72 ms (61.61 ± 4.79) with an interval of 2–5 ms (3.24 ± 0.97). First syllable in these couples lasts 23–33 ms (28.23 ± 1.93) and contains a quieter beginning part (6–8 ms (7.17 ± 0.77)) and a louder part (17–22 ms (21.52 ± 2.33)). First syllable in these couples is followed by a second syllable (except first syllable). Duration of the second syllable varies between 32–42 ms (34.85 ± 2.33).

#### Pezodrymadusa lata

Karabağ, 1961

http://species-id.net/wiki/Pezodrymadusa_lata

##### Distribution.

**Figure 10.**
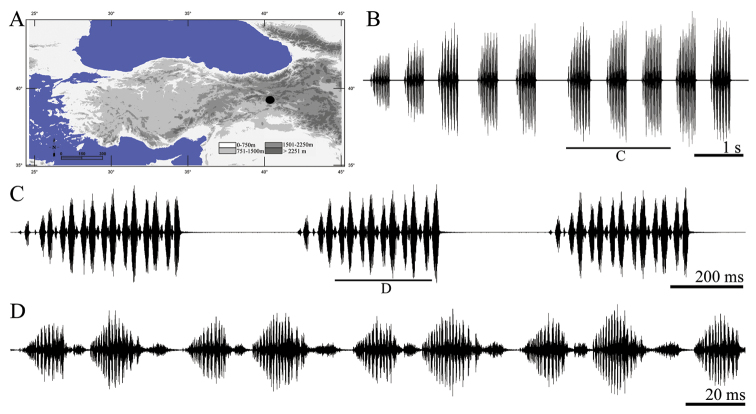
Distribution map (**A**) and male calling song of *Pezodrymadusa lata* (**B** sequences of phrases groups **C** three complete phrase and **D** a group of syllable couples).

Endemic for Turkey – East Anatolia (Figure 10a) (Karabağ 1961).

##### Song recording.

Male specimens collected from Turkey, Malatya, Doğanşehir, Çığlık, 1791 m, 4.VII.2012 (by D. Şirin & A. Mol), and calling song recorded from one male at 30 °C in the field (by A. Mol).

##### Description of song.

A total ofsix records from one male were examined. The calling song consists of a series of regular phrases (Figure 10b) with an interval of 262–604 ms (332 ± 0.11). Phrases are consisting of 11–15 (12.84 ± 0.83) syllables and the number of syllables in 100 ms is approximately two. The phrases begin with a quiet (low amplitude) syllable (Figure 10c). Oscillographic analyses showed that each phrase involves a few couples of syllables (Figure 10d). Syllable couple duration varies between 46–54 ms (50.85 ± 4.79) with an interval of 3–5 ms (4.74 ± 0.77). First syllable in these couples lasts 17–23 ms (21.23 ± 1.88) and contains a louder beginning part (15–18 ms (16.73 ± 1.03)) and a quieter part [3–6 ms (5.02 ± 0.97)]. First syllable in these couples is followed by a second syllable (except first syllable) after an interval of 1–3 ms (1.42 ± 0.11). Duration of the second syllable varies between 28–33 ms (30.15 ± 2.13).

#### Pezodrymadusa subinermis

Karabağ, 1961

http://species-id.net/wiki/Pezodrymadusa_subinermis

##### Distribution.

**Figure 11.**
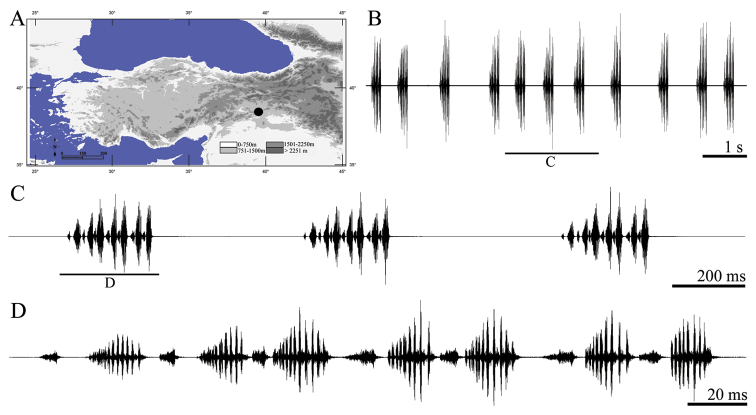
Distribution map (**A**) and male calling song of *Pezodrymadusa subinermis* (**B** sequences of phrases groups **C** three complete phrase and **D** a group of syllable couples).

Endemic for Turkey – East Anatolia (Figure 11a) (Karabağ 1961).

##### Song recording.

Male specimens collected from Turkey, Elazığ, Sivrice, Hazarbaba Kayak Merkezi civarı, 38°25.029'N, 39°18.766'E, 1790 m, 3.VII.2012 (by D. Şirin & A. Mol), and calling song recorded from two males at 30 °C in the field which is type locality of species (by D. Şirin).

##### Description of song.

Totally five records from two males were examined. The calling song consists of a series of regular phrases (Figure 11b) with an interval 355–903 ms (567 ± 0.20). Phrases are consisting of 7–9 (7.23 ± 0.63) syllables. The phrases begin with a quiet (low amplitude) syllable (Figure 11c). Oscillographic analyses showed that each phrase involves a few couples of syllables (Figure 11d). Syllable couple duration varies between 54–65 ms (59.85 ± 2.72) with an interval of 3–6 ms (4.97 ± 0.73). First syllable in these couples last 22–27 ms (24.73 ± 1.58) and contain a louder beginning part [15–18 ms (16.67 ± 1.12)] and a quieter part [4–8 ms (6.62 ± 1.07)]. First syllable in these couples is followed by a second syllable (except first syllable) after an interval of 1–3 ms (1.22 ± 0.09). Duration of the second syllable varies between 31 and 37 ms (33.95 ± 2.17) and includes a louder part [19–23 ms (21.95 ± 1.36)] and a pulse like quieter part (except last syllable) with duration of 8–12 ms (10.72 ± 1.43).

#### Psorodonotus davisi

Karabağ, 1956

http://species-id.net/wiki/Psorodonotus_davisi

##### Distribution.

**Figure 12.**
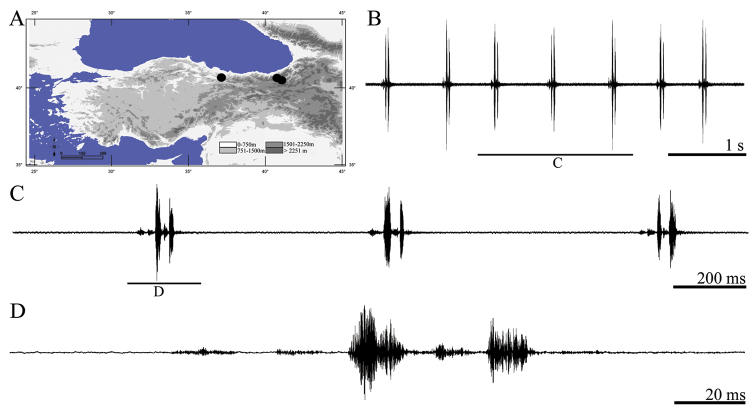
Distribution map (**A**) and male calling song of *Psorodonotus davisi* (**B** sequences of syllables **C** three complete syllables and **D** a complete syllable).

Endemic for Turkey – North East Anatolia (Figure 12a) (Karabağ 1956, Karabağ 1958)

##### Song recording.

Male specimens collected from Turkey, Rize, Ovit Dağı, 1600 m, 20.X.2005, 40°38.626'N, 40°44.234'E, (by A. Mol) and calling song recorded from two males at 24 °C in the field (by A. Mol).

##### Description of song.

Totally six records from two males were examined. The calling song includes rarely one usually several isolated syllables (Figure 12b) with an interval 387–632 ms (526 ± 0.07). Syllable duration varies between 101–117 ms (110 ± 4.21). Oscillographic analyses showed that each syllable follows generally two cycles (Figure 12c, 12d). First cycle lasts 65–71 ms (68.71 ± 2.42) and contains two similar quieter parts (each of 15–20 ms) and a louder part [20–28 ms (24.28 ± 2.57)]. Second cycle of syllables varies between 31–40 ms (36.72 ± 2.23) and includes a pulse like quieter part [8–14 (11.45 ± 2.89)] and a louder part which lasts 16 to 24 ms (18.72 ± 3.12).

#### Psorodonotus ebneri

Karabağ, 1952

http://species-id.net/wiki/Psorodonotus_ebneri

##### Distribution.

**Figure 13.**
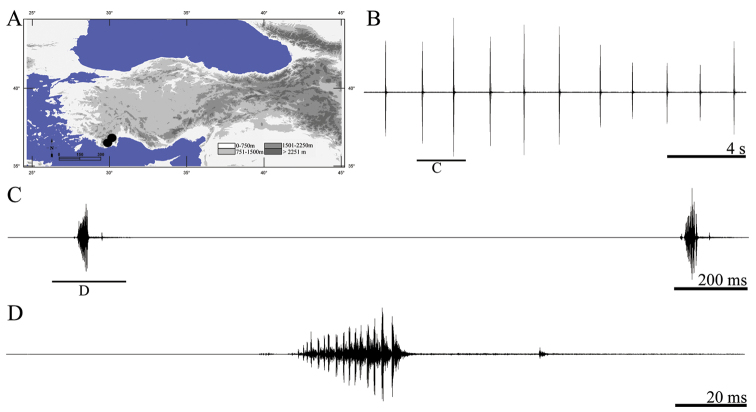
Distribution map (**A**) and male calling song of *Psorodonotus ebneri* (**B** sequences of syllables **C** two complete syllables and **D** a complete syllable).

Endemic for Turkey – Southwest Anatolia (Figure 13a) (Karabağ 1952, Karabağ 1958).

##### Song recording.

Male specimens collected from Turkey, Antalya, Saklıkent, Bakırlıdağ-Pozan arası (It is type locality of species.), 36°49.615'N, 30°17.215'E, 1765 m, 30.VII.2010 (by A. Mol) and calling song recorded from two males at 31 °C in laboratory (D. Şirin).

##### Description of song.

Totally five records from two males were examined. The calling song includes isolated syllables (Figure 13b, c) with an interval 1.76–2.52 s (2.18 ± 0.15). Syllable duration varies between 73–88 ms (80.41 ± 4.96). Oscillographic analyses showed that each syllable includes three elements (quieter-louder-quieter) (Figure 13d). First element of a syllable (rarely absent) is a quieter part and its duration varies between 3–6 ms (3.67 ± 0.29). The first element of syllable is followed by a louder part after an interval of 4–7 ms (5.57 ± 0.81). The louder part (middle element) of syllable consists of 12–18 (15.33 ± 1.96) pulses and its duration varies from 33 to 38 ms (35.26 ± 1.88). The louder part is followed by another quieter part (last element) after an interval of 25–32 ms (27.83 ± 2.40) and its duration varies between 2 and 4 ms (3.10 ± 0.60).

#### Psorodonotus rugulosus

Karabağ, 1952

http://species-id.net/wiki/Psorodonotus_rugulosus

##### Distribution.

**Figure 14.**
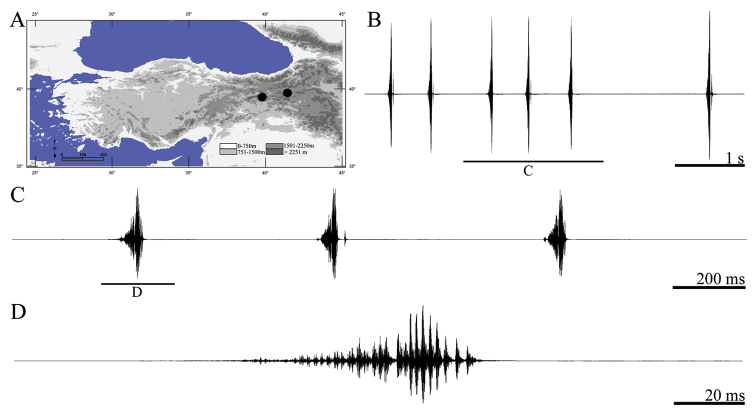
Distribution map (**A**) and male calling song of *Psorodonotus rugulosus* (**B** sequences of syllables **C** three complete syllables and **D** a complete syllable).

Endemic for Turkey – North East Anatolia, East Anatolia (Figure 14a) (Karabağ 1952, 1958, Ünal 2006).

##### Song recording.

Male specimens collected from Turkey, Erzincan, Kelkit-Pöske yolu, Ahmetli çıkışı, 2016 m, 30.VI.2013, 39°53.392'N, 39°21.588'E, (by D. Şirin, A. Mol & M.S. Taylan) and calling song recorded from two males at 28 °C in the field (by D. Şirin).

##### Description of song.

Totally six records from two males were examined. The calling song includes isolated syllables (Figure 14b, c) separated by intervals of 456–1915 ms (833 ± 0.25). Syllable duration varies between 62 and 90 ms (78.83 ± 4.68). Oscillographic analyses showed that each syllable includes generally two elements (quieter and louder) (Figure 14d). First element of a syllable (rarely absent) is a quieter part and its duration varies between 5 and 6 ms (5.77 ± 0.19). The first element of syllable is followed by a louder part with an interval of 4–6 ms (5.27 ± 0.62). The louder part (second element) of syllable consists of 24–32 (27.63 ± 3.13) pulses and its duration lasts from 48 to 56 ms (51.21 ± 2.14). Sometimes the louder part is followed by another quieter part with an interval of 13–19 ms (16.03 ± 2.24) and duration of 4–8 ms (5.20 ± 0.72) (Figure 14c).

### Bradyporinae Burmeister, 1838

#### Bradyporus (Callimenus) avanos

Ünal, 2011

http://species-id.net/wiki/Bradyporus_avanos

##### Distribution.

**Figure 15.**
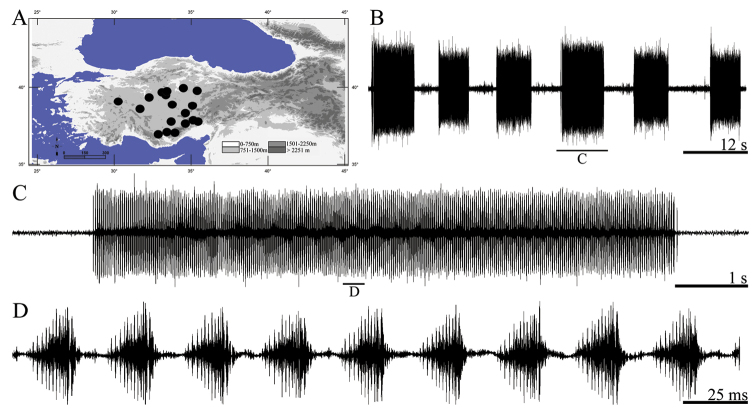
Distribution map (**A**) and male calling song of *Bradyporus (Callimenus) avanos* (**B** a group of sequences of syllables **C** a complete sequences of syllables and **D** a group of syllables).

Endemic for Turkey, widespread in central Anatolia (Figure 15a) (Ünal 2011).

##### Song recording.

Male specimens collected from Turkey, Tokat, Çamlıbel, Artova yol ayrımı, 40°09.680'N, 35°54.309'E, 1280 m, 17. VII. 2004, (by A. Mol) and calling song recorded from two males at 32.8 °C in the field (by A. Mol).

##### Description of song.

Total of five records from two males was examined. The calling song consists of polysyllabic sequences of variable duration (Figure 15b) with an interval of 5.80–8.02 s (mean 6.82). Sequences are consisting of 215–350 (262 ± 38.76) syllables. Nearly all syllables are in same amplitude (Figure 15c); rarely syllables in begin or end point of sequences are in low amplitude. General syllables shape is a kind of crescendo (Figure 15d). Syllable period durations vary between 21 and 28 ms (25.68 ± 1.89). The number of syllables in 100 ms is approximately four (Figure 15d). Each syllable includes 14–23 impulses (16 ± 2.14).

### Saginae Brunner von Wattenwyl, 1878

#### Saga cappadocica

Werner, 1903

http://species-id.net/wiki/Saga_cappadocica

##### Distribution.

**Figure 16.**
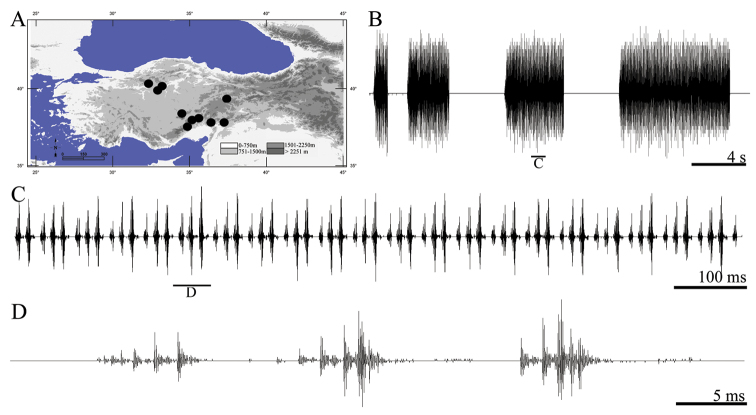
Distribution map (**A**) and male calling song of *Saga cappadocica* (**B** sequences of repetitive units **C** a group of repetitive units and **D** a complete repetitive unit).

Endemic for Turkey – central Anatolia (Figure 16a) (Kaltenbach 1967, 1970).

##### Song recording.

Male specimens collected from Turkey, Ankara, Çubuk-Şabanözü yolu 6 km, Mutlu köyü yolu, 40°14.760'N, 33°05.199'E, 1090 m, 10.VII.2011 (by D. Şirin) and calling song recorded from 3 males at 24 °C in the field (by D. Şirin).

##### Description of song.

Total of six records from three males was examined. Oscillographic analyses showed that two possibilities (i) each syllable contains three similar elements (usually crescendo) and phrase consists of a great number of them or (ii) there are micro-phrases of three syllables in a crescending sequence and phrase consists of a great number of this micro-phrases (Figure 16c). So, repetitive unit term was used for this situation to describe the song. The calling song consists of repetitive unit sequences of variable duration (Figure 16b). The phrase begins with 1–2 repetitive units that are quieter than the following ones. Phrase duration varies between 1.02 and 8.12 s (4.15 ± 1.29). The number of repetitive unit in 100 ms is approximately 2.5 and repetitive unit duration varies between 36 and 42 ms (39.11 ± 1.90) with an interval of 6–9 ms (6.72 ± 0.19). Each element includes 4–8 impulses and the duration of each element (Figure 16d) varies between 5 and 9 ms (7.16 ± 0.79).

#### Saga rhodiensis

Salfi, 1929

http://species-id.net/wiki/Saga_rhodiensis

##### Distribution.

**Figure 17.**
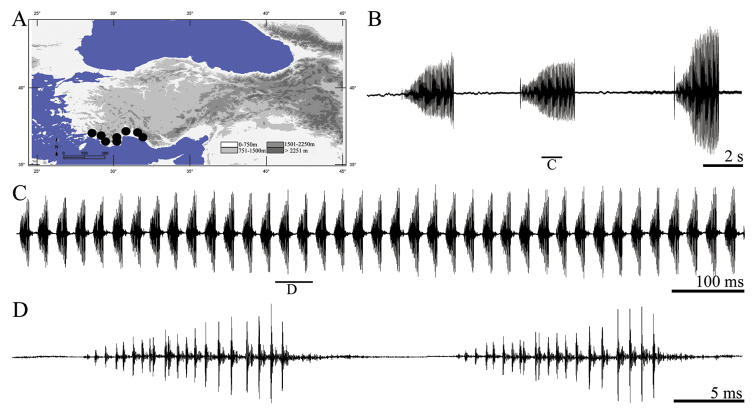
Distribution map (**A**) and male calling song of *Saga rhodiensis* (**B** sequences of phrases groups **C** a group of syllable sequences and **D** two complete syllable).

Anatolia and Rhodos (Figure 17a) (Kaltenbach 1967, 1970).

##### Song recording.

Male specimens collected from Turkey, Antalya, Kemer, Tahtalı Dağları, Gürleyik mevkii, 36°33.067'N, 30°25.001'E, 1479 m, 2.VIII.2010 (by D. Şirin & U. Şirin) and calling song recorded from two males at 31 °C in the field (by D. Şirin).

##### Description of song.

Total of five records from two males was examined. The calling song consists of regular phrases (Figure 17b) with an interval of 3.25–4.50 s. The phrase begins with characteristic high amplitude syllable in the all phrases. After this syllable phrases continue with a quiet beginning and maximum intensity is usually reached between 1/4 and 1/3 of the phrase, however, in some of the phrases there is often a more gradual crescendo roughly up to half of the phrase (Figure 17c). Phrases contain 86–103 syllables (94.2 ± 7.02) and duration varies between 2.27 and 2.77 s (2.55 ± 2.54). Amplitude of the impulses of each syllable from beginning to end of it is getting louder (Figure 17d). Therefore general song shape shows crescendo type (Figure 17c, 17d). The number of syllables in 100 ms is 4–5 (4.18 ± 0.19). Syllable duration varies between 14 and 28 ms (20.92 ± 1.79) with an interval of 3–7 ms (5.62 ± 0.11). Oscillographic analyses showed that each syllable consists of a single element and includes easily countable impulses 16–22 (19.72 ± 1.87) in a crescending structure (Figure 17d).

## Discussion

This study is the first one to reveal the descriptions of the acoustic parameters (amplitude-temporal pattern) of 14 endemic and two sub-endemic species in Anatolia. Also, this data could be used as an archive to determine the species in the field (Oliveira et al. 2001) which is important for species having local distribution in nature, among which the endemics studied herewith.

**Remarks on song patterns:** This part of our discussion focuses only on *Parapholidoptera*, *Pezodrymadusa*, *Psorodonotus* and *Saga*, because we posses sufficient amount of comparative data only on these genera. The four species of genus *Pezodrymadusa* show very similar song patterns, similar to the song pattern in genus *Eupholidoptera* (Heller 2006, Çıplak et al. 2009). *Eupholidoptera* is a well known genus and all the species in the genus have uniform song pattern, but a different morphology (Heller 2006, Çıplak et al. 2009). All four species, *Pezodrymadusa konowi* (Bolivar, 1899), *Pezodrymadusa kurmana* (Ramme, 1939), *Pezodrymadusa lata* Karabağ, 1961, and *Pezodrymadusa subinermis* Karabağ, 1961, produce a multi-syllable song with syllable groups (Figures 8b, 9b, 10b, 11b). Within these four species, *Pezodrymadusa subinermis* has the lowest syllable number (Figures 11b–c), whereas *Pezodrymadusa lata* has the highest syllable number in a phrase (Figures 10b–c). *Pezodrymadusa subinermis* shows partially differences in the fine structure of syllables than the other species in this study.

*Parapholidoptera* is the second species-rich genus of the tribe Pholidopterini (including *Pholidoptera*, *Eupholidoptera*, *Apholidoptera*, *Uvarovistia*, *Parapholidoptera*, *Exopholidoptera*) in Anatolia (Çıplak 2000, Çıplak et al. 2002, Eades et al. 2013). Genus *Parapholidoptera* was studied morphologically by Çıplak (2000) and acoustically by Heller (2006). Songs of six species have already been described in the *Parapholidoptera castaneoviridis* and *Parapholidoptera distincta* groups (Heller 2006). Song records of three *Parapholidoptera* species are the members of the *Parapholidoptera castaneoviridis* group according to cladograms obtained based on the morphological data (Çıplak 2000). Heller (2006) indicated that only *Parapholidoptera salmani* presumably has a differentiated song pattern within *Parapholidoptera castaneoviridis* group. However, the results of this study show that the general song pattern of *Parapholidoptera salmani* is similar to the *Parapholidoptera castaneoviridis* group song pattern (Figures 7b–c). On the other hand, *Parapholidoptera castaneoviridis* group syllable pattern consists of coupled pulses; however, thesyllables of *Parapholidoptera salmani* song consist of one continuous impulse series without any interval (Figure 7d).

Genus *Psorodonotus* has 11 species and eight of them are endemic/subendemic to Anatolia (especially north-east Anatolia) (Çıplak 2008, Ünal 2013a, Eades et al. 2013). The recorded song data in this study show interspecific differences. According to the song results in this study, *Psorodonotus ebneri* Karabağ, 1952 and *Psorodonotus rugulosus* Karabağ, 1952 exhibit a similar song pattern (Figures 13c–d, 14c–d). The song of *Psorodonotus davisi* Karabağ, 1956 shows different syllable composition (Figure 12). However, the song pattern indicates a close relationship between *Psorodonotus ebneri* and *Psorodonotus rugulosus*, although they are not close geographically. On the other hand, *Psorodonotus davisi* and *Psorodonotus rugulosus* are located close to each other and far away from the *Psorodonotus ebneri*.

The saw-legged bush-crickets are among the largest insect species in the Palaearctic. The range of most species of this genus covers the Balkan Peninsula and Asian Turkey (Kaltenbach 1967, 1970). Seven of these species are found in Asian Turkey (*Saga beieri*, *Saga cappadocica*, *Saga longicaudata*, *Saga ephippigera*, *Saga natoliae*, *Saga puella* and *Saga rhodiensis*) (Karabağ 1958, Çıplak et al. 2002). Five European taxa (*Saga campbelli*, *Saga gracilis*, *Saga helenica*, *Saga rammei* and *Saga natoliae*) were discussed in detail using songs characteristics (Kolics et al. 2008). The songs of *Saga rhodiensis* Salfi, 1929 and *Saga cappadocica* Werner, 1903 are described in this study. The song of *Saga rhodiensis* shows a similar song pattern with these five species, but differs in length and impulse number of the syllables from them. On the other hand, *Saga cappadocica* shows distinct syllable elements composition (Figures 16b–d, 17b–d). According to the results of this study and Kolics et al. (2008), the song patterns in genus *Saga* are distinct between taxa and they could be used for taxonomic purposes.

**Remarks on the relation of distribution and song diversity:** Genus *Pezodrymadusa* is distributed in Anatolia with 14 endemic taxa, in Caucasia with *Pezodrymadusa magnifica*, and in Iran with *Pezodrymadusa grisea* (Eades et al. 2013). Anatolian species are distributed in a narrow area separated by short distances especially in the eastern part of central Anatolia and the eastern Anatolia (Karabağ 1961, Sevgili et al. 2012b, Ünal 2013b). This distribution gives us a hint on why the species of this genus have a uniform-like song pattern but a different morphology. Heller (2006) mentions a similar situation in different allopatric groups or genera, such as *Psorodonotus fieberi* ssp. or *Eupholidoptera*, and suggests as the most possible explanation the fact that the changes in song in these groups appear slower than the changes in morphology. Similarly, *Pezodrymadusa* shows the same pattern for the recorded taxa.

*Parapholidoptera castaneoviridis* species-group has 16 members (Eades et al. 2013). Up to now, the songs of the four species of this group have been described showing identical pattern (Heller 2006). The song pattern of the three species described in the present study and belonging to the same species-group, also corresponds to the latter. The general distribution of the species in genus *Parapholidoptera* shows allopatric pattern, but only *Pezodrymadusa distincta* and *Pezodrymadusa signata* occur parapatrically (Heller 2006). These two parapatric species have a very different song pattern (see detail in Heller 2006). When the distribution and song diversity of *Parapholidoptera* species are considered, parapatric taxa develop stronger acoustic specializations than allopatric taxa.

However, though in *Parapholidoptera castaneoviridis* species-group and genus *Pezodrymadusa* “*changes in song appear more slowly than changes in morphology*” (Heller 2006), genus *Psorodonotus* tells us a different story. The distribution of *Psorodonotus* shows in general an allopatric pattern, but *Psorodonotus davisi* and *Psorodonotus specularis* occur parapatrically (Karabağ 1958, Ünal 2006, Ünal 2013b, unpublished data of Deniz Şirin). Heller (2006) shows that the three subspecies of *Psorodonotus fieberi* songs do not differ. However, the song diversity of *Psorodonotus* species recorded in this study discloses that the song is applicable to species identification. Besides, the males of the studied species also differ in matter of comparison with the titilators (Karabağ 1952, Karabağ 1956). These data about *Psorodonotus* appear in the case of “allopatric forms differ in song and also morphology”.

## Supplementary Material

XML Treatment for Anterastes tolunayi

XML Treatment for Apholidoptera pietschmanni

XML Treatment for Gampsocleis recticauda

XML Treatment for Parapholidoptera bolkarensis

XML Treatment for Parapholidoptera intermixta

XML Treatment for Parapholidoptera salmani

XML Treatment for Pezodrymadusa konowi

XML Treatment for Pezodrymadusa kurmana

XML Treatment for Pezodrymadusa lata

XML Treatment for Pezodrymadusa subinermis

XML Treatment for Psorodonotus davisi

XML Treatment for Psorodonotus ebneri

XML Treatment for Psorodonotus rugulosus

XML Treatment for Bradyporus (Callimenus) avanos

XML Treatment for Saga cappadocica

XML Treatment for Saga rhodiensis
